# Neutrinos to Astrovirology: Signatures

**DOI:** 10.6026/973206300200018

**Published:** 2024-01-31

**Authors:** Paul Shapshak, Milad Zandi, Charurut Somboonwit, John T Sinnott

**Affiliations:** 1 Department of Internal Medicine, Morsani College of Medicine, University of South Florida, Tampa, Florida 33606, USA; 2 Hepatitis Research Center, Department of Virology, Faculty of Medicine, Lorestan University of Medical Sciences, Khorramabad, Iran

**Keywords:** Biosignatures, biodefense, exoplanets, astrobiology, astrovirology, terrestrial, unity life, molecular biology, biochemistry, communication, Extra-Terrestrial Intelligence (ETI), neutrino, electromagnetic radiation, Sagan-Kardashev, Dyson, goldilocks, entropy, space exploration, contamination from Earth, extra-terrestrial biosafety zone, NIH, CDC, WHO

## Abstract

In the 20th century, the concept of terrestrial life's unity was solidified, and the 21st century saw the emergence and establishment of astrovirology. To date, life originating beyond Earth has not been identified. The singular instance where NASA investigated potential microfossils in Martian ejecta found on Earth has since been refuted. This report suggests that a more comprehensive discussion and analysis of life's biosignatures and communication methods are essential. Such approaches are crucial not only to avoid overlooking the possible existence of extra-terrestrial intelligence (ETI) but also to prevent potential human infections that could arise from extra-terrestrial contact. In addition terrestrial infections by microorganism that originally derived from Earth and were returned, require investigation due to potential mutations and subsequent increased pathogenicity.

## Bio signatures:

In 2018, Schwieterman *et al.* provided a comprehensive review of potential biosignatures on exoplanets, exploring various indicators that might be employed to detect extra-terrestrial life [1]. Concurrently, Stedman *et al.* advocated for Astrovirology, investigating the possibility of viruses coexisting with life in extra-terrestrial environments [2]. The alleged cellular fossils discovered within Martian ejecta on Earth, as identified by NASA, remain contentious and have largely been dismissed [3, 4]. As a result, the assertion stands that life originating from outside Earth has not yet been confirmed. However, these reviews and wider literature often presuppose goldilocks conditions as a fundamental requirement for life. This concept has sparked much debate. This narrow focus on terrestrial-like habitable zones necessitates a reevaluation. The search for biosignatures should be broadened to encompass diverse environments and chemical and biochemical systems, which are not terrestrial-like.

In expanding this search, it is reasonable to consider an array of astrobiological interactions. For instance, the possibility of extra-terrestrial intelligence in the Milky Way and other galaxies, potentially communicating through neutrino signals in addition to electromagnetic radiation, should be explored. This concept, including laboratory experiments on neutrino signal transmission through the Earth, has been discussed in scientific discourse [5,6, 7]. Such innovative approaches could enhance our capacity to detect and understand life beyond our planet.

## Biodefense:

The earlier history of epidemics and pandemics demonstrate general under-preparedness globally for such catastrophes. However, much work has been accomplished by NIH, CDC, and WHO in biomedical research, technology, and organizational skills since the 1918 pandemics [7,8,9]. Biodefensive measures are required for the possibility of remote astrobiological and astrovirological interactions. Additionally, biodefense is needed to broach dangers impelled by off-world evolution of microorganisms originating terrestrially.

## Conclusion:

Paradigm shifts from goldilocks models are needed. Creatively, for example, entropy measures could be used to classify elements and biochemicals rather than the serendipity of terrestrial goldilocks zone biochemistry [10]. Hypothesis changes are required if we are not to miss forms of life and intelligence including Sagan-Kardashev and Dyson civilizations. Likely many will be totally foreign to standard terrestrial biochemical models [5- 7,11].
